# A Diffusion Model to Quantify Membrane Repair Process in *Listeria monocytogenes* Exposed to High Pressure Processing Based on Fluorescence Microscopy Data

**DOI:** 10.3389/fmicb.2021.598739

**Published:** 2021-05-13

**Authors:** Bahareh Nikparvar, Alicia Subires, Marta Capellas, Manuela Hernandez-Herrero, Peter Crauwels, Christian U. Riedel, Nadav Bar

**Affiliations:** ^1^Department of Chemical Engineering, Norwegian University of Science and Technology, Trondheim, Norway; ^2^Department of Animal and Food Science, Autonomous University of Barcelona, Barcelona, Spain; ^3^Department of Biology, Institute of Microbiology and Biotechnology, Ulm University, Ulm, Germany

**Keywords:** high pressure processing, *Listeria monocytogenes*, mathematical modeling, membrane damage, repair process, fluorescence microscopy

## Abstract

The effects of environmental stresses on microorganisms have been well-studied, and cellular responses to stresses such as heat, cold, acids, and salts have been extensively discussed. Although high pressure processing (HPP) is becoming more popular as a preservation method in the food industry, the characteristics of the cellular damage caused by high pressure are unclear, and the microbial response to this stress has not yet been well-explored. We exposed the pathogen *Listeria monocytogenes* to HPP (400 MPa, 8 min, 8°C) and found that the high pressure created plasma membrane pores. Using a common staining technique involving propidium iodide (PI) combined with high-frequency fluorescence microscopy, we monitored the rate of diffusion of PI molecules into hundreds of bacterial cells through these pores on days 0, 1, 2, 3, and 4 after pressurization. We also developed a mathematical dynamic model based on mass transfer and passive diffusion laws, calibrated using our microscopy experiments, to evaluate the response of bacteria to HPP. We found that the rate of diffusion of PI into the cells decreased over the 4 consecutive days after exposure to HPP, indicating repair of the pressure-created membrane pores. The model suggested a temporal change in the size of pores until closure. To the best of our knowledge, this is the first time that pressure-created membrane pores have been quantitatively described and shown to diminish with time. In addition, we found that the membrane repair rate in response to HPP was linear, and growth was temporarily arrested at the population level during the repair period. These results support the existence of a progressive repair process in some of the cells that take up PI, which can therefore be considered as being sub-lethally injured rather than dead. Hence, we showed that a subgroup of bacteria survived HPP and actively repaired their membrane pores.

## 1. Introduction

Bacteria in nature are exposed to various environmental stresses, including changes in temperature or pH, radiation, antimicrobial compounds, and osmotic pressure. Pressurization of bacteria that normally grow in atmospheric conditions may trigger response mechanisms that enable them to adapt to the new pressure condition and survive. Although the cell envelope and particularly the membrane structure have been reported to be susceptible to high pressure processing (HPP) (Pagán and Mackey, 2000; Winter and Jeworrek, 2009; Gänzle and Liu, 2015), the mechanisms that bacteria activate as a response to this stress are still largely unknown. Moreover, the potential existence of a membrane repair machinery in bacteria that responds to pressure-induced damage has not been well-investigated. This phenomenon is of particular importance in the food industry, where exposure to HPP is used as a preservation method to inactivate foodborne bacteria.

Release of low-molecular-weight metabolites, including nucleotides, amino acids, and inorganic ions, from bacterial cells exposed to different types of stress such as antibiotics or bacteriocins has been proposed as an indicator of membrane damage (Lambert and Hammond, 1973; Gilbert et al., 1977; Broxton et al., 1983; Zhen et al., 2013; Singh et al., 2016). Two decades ago, high pressure stress had already been shown to cause deformation of the cell membrane and create surface pores (Ritz et al., 2001). Several authors demonstrated increased uptake of exogenous fluorescent molecules by pressurized cells. For example, membrane damage in *Lactobacillus plantarum* exposed to high pressure was shown by staining cells with the fluorescent dye propidium iodide (PI) (Smelt et al., 1994). In the same work, leakage of ATP from these cells was observed, indicating a leaky membrane (Smelt et al., 1994). Gänzle and Vogel (2001) showed changes in the kinetics of outer and cytoplasmic membrane permeability in *Escherichia (E.) coli* after exposure to high pressure (300, 500, 600 MPa) by staining of treated cells with PI and 1-N-phenylnaphthylamine. Furthermore, a few studies have detected cellular proteins outside the cell after exposure to high pressure (Smelt et al., 1994; Gänzle and Vogel, 2001; Mañas and Mackey, 2004). One such example is the study of Mañas and Mackey (2004), which detected intracellular proteins outside pressure-treated *E. coli* cells (200 MPa, 8 min), indicating membrane leakage.

Several previous experiments showed that pressure-mediated damage in bacteria could be repairable such that the cells could potentially grow after repair of the site of injury during storage (Bozoglu et al., 2004; Jofré et al., 2010; Klotz et al., 2010). Bozoglu et al. (2004) observed no colony formation in selective or non-selective agar immediately after pressure treatment at 550 MPa, suggesting that all cells were inactivated. However, they detected growth in both selective and non-selective agar after 6 days at 4°C, and after 1 day at 22 and 30°C, probably due to a recovery process. Jofré et al. (2010) reported that even after a high pressure treatment of 900 MPa, some *Listeria (L.) monocytogenes* cells remained viable and were able to recover at 14°C. Klotz et al. (2010) examined the susceptibility of cell membranes in *E. coli* to pressure-induced damage (500, 600, and 700 MPa) and found that in a pressure-resistant strain, uptake of PI occurred only during exposure but not after pressure release, indicating that the cells were able to reseal their leaky membranes. This again supports the hypothesis of the presence of a recovery process in sub-lethally injured cells, and adds to the evidence in the literature that, in contrast to what has been traditionally assumed, cells that take up PI are not always dead (Shi et al., 2007; Davey and Hexley, 2011; Subires et al., 2013; Yang et al., 2015).

Several authors have proposed simple models to describe inactivation of pressure-treated bacteria and their growth behaviors (Koseki et al., 2007; Bover-Cid et al., 2010; Hereu et al., 2014; Valdramidis et al., 2015; Rubio et al., 2018). However, although a few of these studies reported the existence of a repair process, they did not identify the underlying mechanisms that allow bacteria to recover.

Here, we focused on foodborne pathogenic bacteria *L. monocytogenes* and the effects of HPP (400 MPa, 8 min, 8°C) on its membrane. We developed a dynamic model that could estimate pressure-induced membrane damage over time.

Our predictions and findings suggest that sub-lethally injured pressurized cells were able to repair their membranes by resealing the surface pores after decompression. To the best of our knowledge, this is the first time that the repair of membrane pores following HPP has been shown in bacteria.The estimated time required for resealing the pore area could be useful for the food industry to adjust the high pressure strategy applied (particularly by adjusting the pressure strength and holding time) to design a more effective food preservation process.

## 2. Materials and Methods

### 2.1. Experiments

#### 2.1.1. Bacterial Strain and Culture Conditions

The *L. monocytogenes* Scott A strain used in this study was provided by the Collection of Institut Pasteur (CIP 103575; Paris, France) and selected based on its increased high pressure resistance and widespread use as a reference strain in food preservation technology testing (Alpas et al., 1999; Briers et al., 2011; Duru et al., 2020). Stock cultures grown in tryptone soya broth supplemented with 0.6% w/v yeast extract (TSBYE; Oxoid/ThermoFisher Scientific, Hampshire, UK) were stored at −80°C in glycerol (33% v/v). A loopful of the glycerol stock was inoculated into 20 mL TSBYE and incubated at 37°C overnight in a shaking (80 rpm) water bath. To prepare working cultures in the early stationary phase, the overnight culture was diluted to 1:100 in 20 mL of fresh TSBYE and incubated under the same conditions for 18 h. The time to reach this growth phase was established by monitoring the optical density at 600 nm (Bioscreen C; Oy Growth Curves AB, Helsinki, Finland) in a separate experiment.

#### 2.1.2. High Pressure Processing (HPP)

Early stationary phase cultures were transferred to 30-mL HDPE bottles with screw caps (BNH0030PN, SciLabware Limited, Stoke-on-Trent, UK), avoiding the presence of air bubbles. Bottles had previously been sterilized with a 70% v/v ethanol solution overnight, rinsed three times with sterile distilled water, and dried at 60°C in an incubator. The possible presence of ethanol residues after this procedure was ruled out, as growth of untreated samples was not reduced (data not shown). To prevent hazardous culture spills, caps were sealed with laboratory film, and bottles were vacuum packed in sterile plastic bags. Samples were then cooled to 8°C in ice before HPP (approximately 30 min).

HPP was performed in a discontinuous isostatic press (Alstom ACB, Nantes, France) fitted with a 2-L pressure chamber containing water. The temperature of the pressure chamber and transmission fluid was adjusted to the treatment temperature (8°C) using an external continuous cooling system. The samples were then placed inside the chamber, allowed to re-equilibrate to 8°C for 5 min, and pressurized at 400 MPa for 8 min. The temperature was selected according to the common HPP conducted in the industry (Muntean et al., 2016). The come-up time and the decompression time were both 2 min. Pressurized samples were kept in ice before being further processed for fluorescence microscopy analysis, and then stored at 8°C for the subsequent days of analysis. The storage temperature was selected to simulate the temperature deviations that occur in the cold chain/storage of refrigerated food products, i.e., abuse temperature (Alvarez-Ordóñez et al., 2015).

#### 2.1.3. Cell Preparation and Fluorescence Microscopy Analysis

Pressure-treated cells were pelleted (13,000 g, 1 min) and resuspended in Dulbecco's phosphate-buffered saline (DPBS, pH 7.4) immediately before microscopy analysis at 1 h (day 0), and on days 1, 2, 3, and 4 post-treatment. Sixty microliters of this cell suspension was pipetted into the center of a glass-bottomed microwell plate (35 mm petri dish, 14 mm microwell, coverglass No. 1.5; MatTek Corporation, Ashland, USA). To immobilize cells and provide a stationary frame for real-time imaging, 200 μL of a 2% w/v low-gelling-temperature agarose (Sigma-Aldrich, Saint Louis, USA) solution was carefully dispensed over the cell suspension drop, first surrounding and then covering it. Approximately 2 mL of a staining solution containing 1.25 μM PI (final concentration; 20 mM stock solution in DMSO, Life Technologies/ThermoFisher Scientific, Darmstadt, Germany) in DPBS was carefully dispensed over the cell suspension and agarose mixture during real-time imaging. The delivery system consisted of a syringe coupled to a plastic tube that was in turn attached to a holed plate lid. Untreated and heat-treated (80°C for 40 min) cell suspensions were prepared as described above and used as a negative and a positive control for PI staining, respectively.

Time-lapse image acquisition was performed with a Leica TCS SP5 (Leica Microsystems, Wetzlar, Germany) confocal microscope using a 63x oil objective (NA 1.4), filters for PI detection (excitation at 575–625 nm; emission at 660–710 nm), and a hybrid detector. For each sample and field, images were captured for 30 min at 1 frame per second (fps). The maximum achievable fluorescence intensity (FI) was obtained from the heat-treated sample, which was used every day as the reference to set the microscope gain parameter.

#### 2.1.4. Culture-Based Cell Counting

The number of viable *L. monocytogenes* cells was determined by the spread plate count method before exposure to HPP (untreated) and on days 0, 1, 2, 3, and 7 after the treatment (400 MPa, 8 min, 8 °C). Samples were serially diluted at the designated time points in peptone saline solution (1 g/L neutralized bacteriological peptone [Oxoid/ThermoFisher Scientific] and 8.5 g/L NaCl in water). Appropriate dilutions were spiral-plated (Eddy Jet; IUL, Barcelona, Spain) on the non-selective medium tryptone soya agar supplemented with 0.6% (w/v) yeast extract (Oxoid/ThermoFisher Scientific). Plates were then incubated at 37°C for 48 h before colony counting. The limit of detection (LOD) and the limit of quantification (LOQ) (i.e., the lower limit of acceptably accurate cell counts) of this method were 1.00 and 2.40 log CFU/mL, respectively.

### 2.2. Post-processing of Microscopy Data

We used Fiji software, RRID:_SCR_002285 (an image-processing package based on ImageJ), RRID:_SCR_003070, and MATLAB, RRID:_SCR_001622 to derive the mean red FI value for each cell in every single frame of the image stacks (images were captured for 30 min at 1 fps as described above). On each consecutive day, we monitored several fields of view (FOV) such that the total numbers of PI-positive cells (*n*) studied on days 0–4 were 118, 49, 21, 44, and 27, respectively. We analyzed a total of 318 bacteria in 30 FOV during the 4 days. The lowest and the average number of bacteria analyzed per day were 20 and 45 cells, respectively. We monitored on average 3 FOV per day and 10 bacteria per FOV.

One reason that the cell number was different on different days was that cells at the edges or those with improper orientation for image processing were ignored. Another reason could have been a reduction in the number of red cells (cells with PI molecules inside) over the 4 days.

The FI curves for each day were grouped into separate clusters based on the rate of PI diffusion into the cell, as the treated sample was not homogeneous in terms of pressure-induced damage. This was because cells might react differently to high pressure; therefore, differences in the degree of damage and ability to repair would lead to differences in the rate of change of FI and in the rate of PI diffusion through pores. We used the k-means algorithm to cluster the FI curves, where the optimal number of clusters (*k*) was determined using the elbow method (Kassambara, 2017). The procedure was as the following: First, we considered the last point of each intensity curve (i.e., FI value at *t* = 30 min) to create a database for each day. Then we used MATLAB, RRID:_SCR_001622 to run the k-means algorithm for different numbers of clusters of the database for each day: *k* = 1, , 2, ..., 10. For each k, we calculated the sum of the squared distance (SSE) between the centroid of a cluster and each member of that cluster. We looked at the SSE as a function of the number of clusters and chose a number of clusters so that adding another cluster did not improve much better the SSE. The mean and standard deviation of the FI for each cluster of cells were calculated separately for each time point.

To normalize the FI values and therefore make them comparable over the 4 days, on each consecutive day we divided the values obtained from the pressure-treated sample by the maximum intensity value achieved from the positive control sample on that day. This normalized FI value was then used to estimate the pore size.

### 2.3. Statistical Analysis

We used MATLAB, RRID:_SCR_001622 to run analysis of variance (one-way ANOVA) and multi-comparison tests to investigate whether the estimated radius for the pore size on each day significantly differed from the values estimated for the other days. In the multi-comparison analysis, two clusters with specific mean values were considered to be significantly different if their intervals were disjoint, and not significantly different if their intervals overlapped. We used the Bonferroni method to calculate the intervals.

Additionally, We ran the Bartlett test of the null hypothesis that the estimated radius over the 4 days comes from normal distributions with the same variance.

We fitted a weighted least square model (regression model) to the estimated pore values on each day where weights were determined according to the number of cells monitored each day. We used MATLAB, RRID:_SCR_001622 command “fitnlm” to fit our linear regression model (*y* = *ax* + *b*, where *a* and *b* are the fitting parameters) to the estimated values.

### 2.4. A Computational Model to Describe Membrane Recovery

We developed a modified version of the model that Zarnitsyn et al. (2008) proposed for the transmembrane diffusion of small molecules through membrane wounds in human cells after sonication. Our proposed model was constructed based on several assumptions.

**Figure 1:**
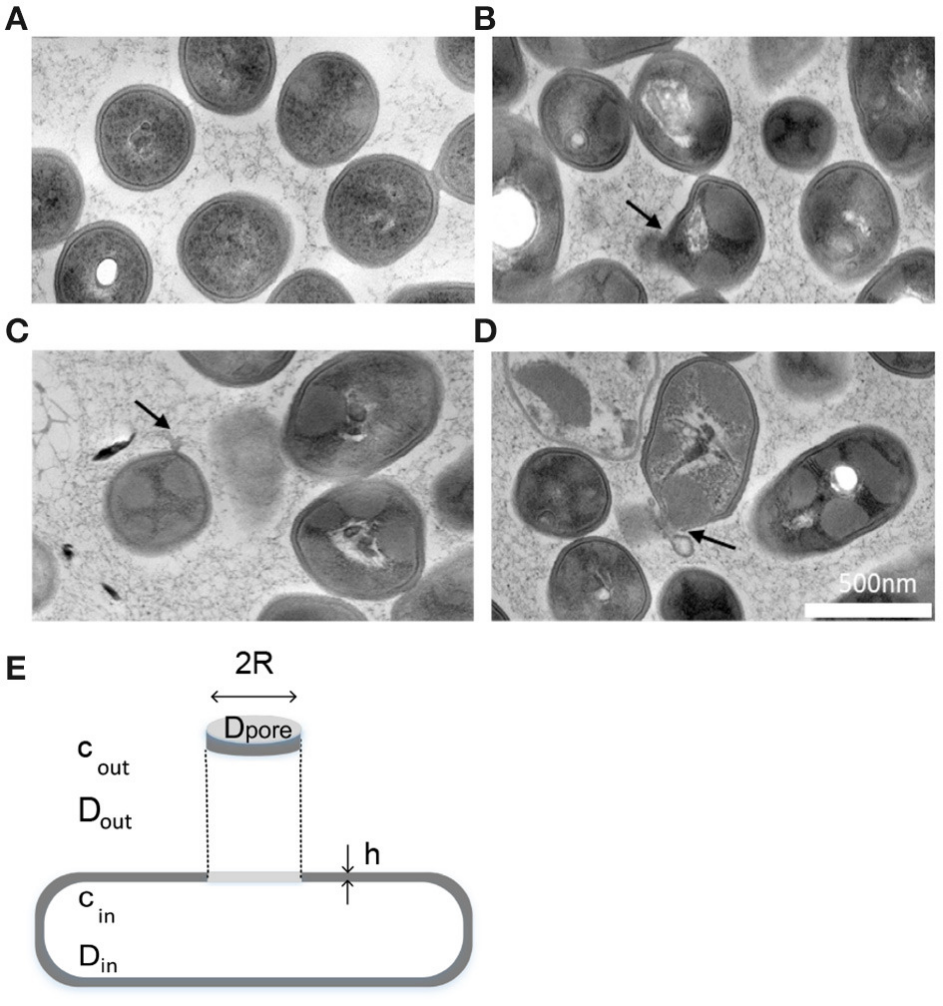
Morphology of pressure-treated cells. **(A–D)** TEM images showed that HPP could create membrane pores (Nikparvar et al., 2019). **(A)** Untreated sample, **(B–D)** Pressure-treated sample, 400 MPa, 15 min. The black arrows show the location of the pore area on the membrane. **(E)** A schematic model showing the morphology of a created membrane pore area under high pressure. The geometry of the bacterium and its plasma membrane with thickness *h* is simplified as a rod shape. *c*_*in*_ and *c*_*out*_ are the concentration of fluorescent molecules inside and outside the cell, respectively, and *D*_*in*_, *D*_*out*_, and *D*_*pore*_ are the diffusion coefficient of the fluorescent molecules inside, outside, and within the pore area, respectively. Symbols are defined in  Table 1.

First, as stated in the literature (Pagán and Mackey, 2000; Winter and Jeworrek, 2009; Gänzle and Liu, 2015) and evidenced by our previous work using flow cytometry (Nikparvar et al., 2019), the bacterial membrane is one of the main structures in the cell that is damaged by HPP. We investigated the morphology of the bacterial membrane after exposure to high pressure using transmission electron microscopy (TEM) and found that the membrane was damaged and became perforated under pressure (Figures 1A–D). We assumed that the main damage occurred in a single pore area. This assumption is valid for our model, because the estimated pore area in our work could be interpreted as an effective area (or total area) regardless of the number of pores. It is mathematically possible to estimate the number of large pores by dividing the effective pore area by the size of the reference molecule (PI), which led us again to one large pore area (for all 4 consecutive days). A schematic geometry for the described membrane pore is shown in Figure 1E.

Second, the membrane, which is otherwise impermeable to fluorescent molecules such as PI, allows the diffusion of these molecules within the pressure-induced membrane pore, provided the size of the pore area is greater than the size of the molecules. We assumed that the mechanism responsible for the movement of PI molecules was diffusion caused by random molecular motion. The rate of PI diffusion into the cell depends on the size of the pore area; therefore, it is possible to estimate the pore size by measuring the difference in FI resulting from the increased number of PI molecules bound to DNA. Based on the mass balance of PI molecules, we can write:

(1) $$\begin{array}{l} V_{cell}\frac{dc_{in}}{dt}=A_{pore}k\left(c_{out}\left(t\right)-c_{in}\left(t\right)\right), \end{array}$$

where *V*_*cell*_ is the cell volume (m^3^); *c*_*in*_ and *c*_*out*_ are the intracellular and extracellular concentration of PI molecules (mol/m^3^), respectively; *t* is time (s); *A*_*pore*_ is the pore area (m^2^); and *k* is the mass transfer coefficient (1/s), which is independent of the pore area and the cell volume but varies with the diffusion coefficient, *D* (m^2^/s), of PI molecules. The expressions on the left and right sides of the equation above (with the unit mol/s) represent the accumulation of PI molecules inside the cell and the rate of the transport into the cell through the pore, *J* (mol/s), respectively. If we assume that PI molecules were added to the sample at *t* = 0, *c*_*in*_(*t* = 0) = 0, and *c*_*out*_(*t* = 0) changes from 0 to *c*_*ou*_*t*__*ss*__, solving Equation (1) gives:

(2) $$\begin{array}{l} c_{in}\left(t\right)=c_{out_{ss}}\left(1-exp\left(-Kt\right)\right), \end{array}$$

where $K=\frac{Ak}{V_{cell}}$. Here, we assume that *A*_*pore*_, *k*, and *V*_*cell*_ are constant during the integrating time, i.e., the period between adding PI molecules to the sample and the time when the FI is saturated (saturation occurred when the intracellular concentration of PI molecules reached the concentration level outside, i.e., *c*_*in*_ = *c*_*ou*_*t*__*ss*__).

According to Equation (2) and by measuring *c*_*in*_ experimentally at time *t*, the quantity *K* can be calculated:

(3) $$\begin{array}{l} K=\frac{1}{t}\ln\left(\frac{1}{1-\frac{I\left(t\right)}{I_{max}}}\right), \end{array}$$

where assuming that the relationship between the concentration of fluorescent molecules and the FI is linear (Kim et al., 2020), we have $\frac{c_{in}\left(t\right)}{c_{{out}_{ss}}}=\frac{I\left(t\right)}{I_{max}}$ (*I*(*t*) and *I*_*max*_ are the intensity at time t and maximum intensity, respectively).

Third, as discussed previously by Zarnitsyn et al. (2008), we considered a three-part process for the diffusion of PI molecules into a cell: diffusion from the extracellular point to the pore; diffusion across the cell membrane within the pore; and diffusion away from the pore in the cytosol. The flow *J* (mol/s) is given by:

(4) $$\begin{array}{l} J=\frac{c_{out}-c_{in}}{\frac{h}{D_{pore}A_{pore}}+\frac{1}{4D_{out}R}+\frac{1}{4D_{in}R}}, \end{array}$$

where *h* is the membrane thickness; *R* represents the pore radius; and *D*_*pore*_, *D*_*out*_, and *D*_*in*_ are the diffusion coefficients of PI molecules inside the pore, outside the cell, and inside the cell, respectively. Symbols are defined in Table 1.

**Table 1:** Definition of symbols.

**Symbol**	**Definition**
*V*_*cell*_ (*m*^3^)	Cell volume
*D*_*out*_ (*m*^2^/*s*)	Extracellular diff. coeff. of PI
*D*_*in*_ (*m*^2^/*s*)	Intracellular diff. coeff. of PI
*D*_*pore*_ (*m*^2^/*s*)	Diff. coeff. of PI inside the pore
*A*_*pore*_ (*m*^2^)	Pore area
*D*_*g*_ (*m*^2^/*s*)	Diff. coeff. of PI in agarose gel
*D*_*w*_ (*m*^2^/*s*)	Diff. coeff. of PI in aqueous solution
*c*_*out*_ (*mol*/*m*^3^)	Extracellular concentration of PI
*c*_*in*_ (*mol*/*m*^3^)	Intracellular concentration of PI
*h* (*m*)	Membrane thickness
*R* (*m*)	Pore radius
*R*_*a*_ (*m*)	Radius of PI molecule
*R*_*g*_ (*m*)	Radius of gel pores
*k*_*B*_ (*J*/*K*)	Boltzmann constant
*T* (*K*)	Temperature
η (*N*.*s*/*m*^2^)	Viscosity of plasma membrane

By substituting $A_{pore}=\pi R^{2}$ (where *R* is the pore radius), *K* can be expressed as a function of *D* and *R*:

(5) $$\begin{array}{l} K=\frac{1}{V_{cell}\left(\frac{h}{D_{pore}\pi R^{2}}+\frac{1}{4D_{out}R}+\frac{1}{4D_{in}R}\right)}. \end{array}$$

As mentioned earlier, we immobilized the cells in agarose gel; therefore, the extracellular diffusion coefficient (*D*_*out*_) was the rate of the diffusion for PI molecules in agarose gel (*D*_*g*_) and equal to σ*D*_*w*_, where *D*_*w*_ is the diffusion coefficient in aqueous solution (Table 2). σ was taken from the literature (Pluen et al., 1999):

(6) $$\begin{array}{l} \sigma =1-2.1444\left(R_{a}/R_{g}\right)+2.08877{\left(R_{a}/R_{g}\right)}^{3}-0.94813{\left(R_{a}/R_{g}\right)}^{5}\\ \;-1.372{\left(R_{a}/R_{g}\right)}^{6}+3.87{\left(R_{a}/R_{g}\right)}^{8}-4.19{\left(R_{a}/R_{g}\right)}^{9}, \end{array}$$

where *R*_*a*_ and *R*_*g*_ are the radii of the PI molecule and gel pore respectively, with the values given in Table 2.

**Table 2:** Constant values.

**Constant**	**Value**	**Reference**
*V*_*cell*_	0.7*10^−18^ (*m*^3^)	For *E. coli* (Yu et al., 2014)
*D*_*w*_	4*10^−10^ (*m*^2^/*s*)	Stokes-Einstein relation^a^
*h*	4*10^−9^ (*m*)	For *E. coli* (Briegel et al., 2009)
*R*_*a*_	6*10^−10^ (*m*)	(Bowman et al., 2010)
*R*_*g*_	8*10^−7^ (*m*)	(Pluen et al., 1999)
*k*_*B*_	1.38*10^−23^ (*J*/*K*)	-
*T*	296 (*K*)	-
η	5*10^−9^ (*N*.*s*/*m*^2^)	(Daniels and Turner, 2007)

a *D_w_ = k_B_T/(6πη_w_R_a_), where η_w_ is the viscosity of water*.

We used the expression that Verkman (2002) introduced to estimate the diffusion coefficient of small molecules (with size and mass approximately equal to those of the PI molecule) in cytoplasm: *D*_*in*_ ≈ 0.25*D*_*w*_. This approximation agrees with the intracellular diffusion coefficient that Zarnitsyn et al. (2008) used to estimate the wound radius in human cell membranes after sonication.

As shown in Figure 1E, the diffusion of fluorescent molecules within the pore can be modeled as diffusion of molecules in a tube of length *h* and radius *R*. We used the expression that Daniels and Turner (2007) proposed for calculating the diffusion coefficient of proteins along long thin membrane tube to express *D*_*pore*_:

(7) $$\begin{array}{l} D_{pore}=\frac{k_{B}T}{4\pi \eta }\left[ln\left(\frac{R}{R_{a}}\right)+O\left(1\right)+O\left(\frac{R_{a}}{R}\right)+...\right], \end{array}$$

where *k*_*B*_ is the Boltzmann constant, *T* is the temperature in Kelvin, η is the viscosity of the membrane, and *R*_*a*_ is the particle (PI) radius (all values in Table 2). O(.) elements are neglected terms from the Taylor series representation. We obtained the measured value for *K* by inserting *I*(*t*) and *I*_*max*_ (Equation 3) and tried to fit our simulated value for *K* (Equation 5) to the measured value for K (Equation 3). By doing so, and by substituting other parameters from the literature and physics of the cells (Table 2), we estimated the parameter *R* in Equation (5) which gave us the pore radius *R*. The time dependent value *K* made the link between our model and experimental data and since we calculated it from the measured data, it specifically affected the final estimation of the pore size, thereby calibrating the model for a specific experimental condition.

Fourth, as we followed an identical protocol for microscopy, it was assumed that the impact of photobleaching (if it occurred) did not interfere with the results.

## 3. Results

### 3.1. Monitoring the Diffusion of PI Molecules Into Damaged Bacterial Cells Through Membrane Pores

**Figure 2:**
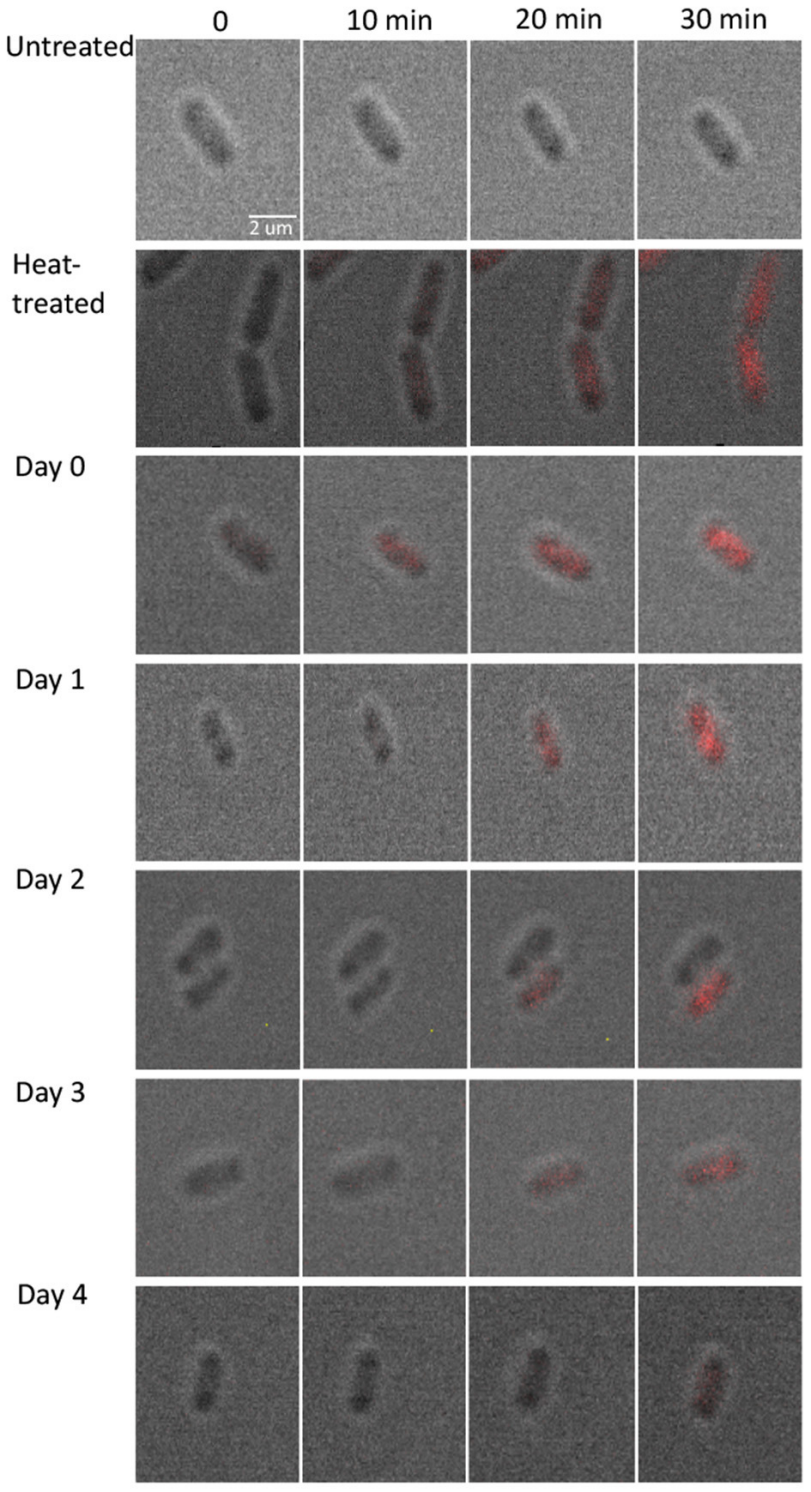
Fluorescence microscopy images for pressure-treated (400 MPa, 8 min, 8°C) and control samples. To measure the diffusion rate of PI molecules through a damaged membrane, we measured red FI for each bacterial cell (*L. monocytogenes*) after adding PI molecules for 30 min at a rate of 1 fps. All images and videos are available from the Dryad Digital Repository (Nikparvar et al., 2020).

**Figure 3:**
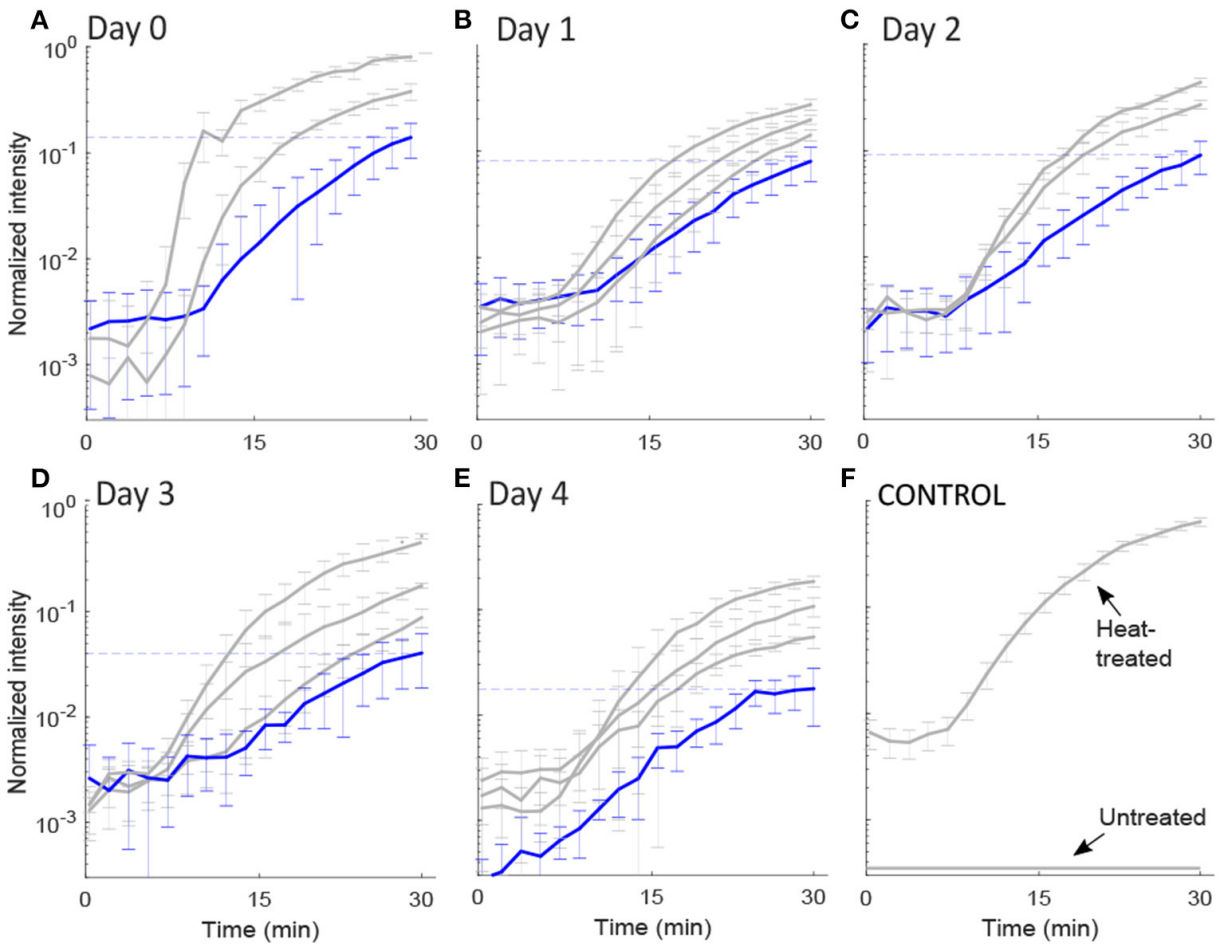
Intensity plots. The normalized mean and standard deviation of the red fluorescence intensity, FI, emitted from: **(A–E)** each pressure-treated cell on days 0–4 following HPP, and **(F)** two control samples. The FI values were extracted from images captured by the fluorescence microscopy technique for 30 min after PI addition. We used the k-means algorithm to cluster the intensity curves through a certain number of clusters (determined by the elbow method) for days 0, 1, 2, 3, and 4 after pressure exposure based on the rate of PI diffusion through membrane pores (see 2.2). The y-axis is in log scale. The intensity value after 30 min for the group of cells with the lowest slope (shown in blue) decreased with the days from 0.14 to 0.02, represented by the dashed lines. Intensity values for each cell (raw data) are available in Supplementary File 1.

To measure the diffusion rate of PI molecules through a damaged membrane, we measured red FI for each bacterial cell (*L. monocytogenes*) after adding PI molecules for 30 min at a rate of 1 fps (Figure 2). All obtained images and videos can be found in the Dryad Digital Repository (Nikparvar et al., 2020). Owing to variability in cell resistance, cells showed different degrees of pressure-induced damage. We used the k-means algorithm to cluster the intensity curves using a certain number of clusters (determined by the elbow method) for days 0, 1, 2, 3, and 4 after pressure exposure based on the rate of PI diffusion through membrane pores (see section 2.2). The mean intensity curve for each cluster on each evaluation day is shown in Figures 3A–E. In the negative control sample (untreated cells), the cells remained uncolored during the observation period, indicating that PI molecules did not diffuse into the cells (Figure 3F). By contrast, in the positive control sample (heat-treated cells), the intensity reached its maximum value after 30 min, indicating substantial membrane damage. The numbers of untreated, heat-treated, and pressure-treated cells in each cluster for days 0–4 are reported in Table 3.

**Table 3:** Number of studied untreated (Untr), heat-treated (H-T), and pressure-treated (PI-positive) cells in each cluster on days 0–4 (D0-D4).

**Cluster**	**D0**	**D1**	**D2**	**D3**	**D4**	**Untr**	**H-T**
Lowest slope	98	12	11	10	7	–	
Mild slope^a^	5	28	3	30	12	–	
Highest slope	15	9	7	4	8	–	
Total	118	49	21	44	27	20	39

a *The number of cells in the mild-slope cluster equals to the number of cells belonging to the clusters other than the lowest- and the highest-slope clusters*.

First, we found that for all evaluation days, the FI started to increase after a delay of about 8 min, probably owing to the time taken for the PI molecules to diffuse from the extracellular medium to the cells through the agarose gel.

Second, Equation (2) implies that the higher the slope of the intensity curve, the faster PI molecules diffuse inside the cell. According to Equations (2) and (5), the diffusion rate of PI molecules (slope of the curves in Figure 3) was positively correlated with the membrane pore size. This suggests that the curves belonging to the cluster with the lowest slope of the intensity curve correspond to the cells with the smallest pore sizes. The intensity curve for the group with the lowest slope displayed an upward trend toward saturation as the time approached 30 min (Figure 3). Conversely, the clusters with the highest slope of the intensity curve correspond to the cells with the largest pore sizes.

Most importantly, we detected a general decrease in the rate of PI diffusion into the cells on consecutive days. This was consistent for the cluster of cells with the lowest rate of diffusion, in which the final FI value (shown with a dashed line in Figure 3) changed from 0.14 on day 0 to a final value of 0.02 on day 4, i.e., a seven-fold decrease. This is a strong indication that the pore size decreased over the 4 days after the pressure treatment.

### 3.2. Estimation of the Pore Area

As discussed earlier, we assumed that the cluster corresponding to the curves with the lowest slope of the intensity curve was associated with the least-damaged bacteria. As we could detect a decay in the rate of PI uptake for this group of cells from day 0 to day 4, we used this group to calibrate the model that estimates the pore size.

**Figure 4:**
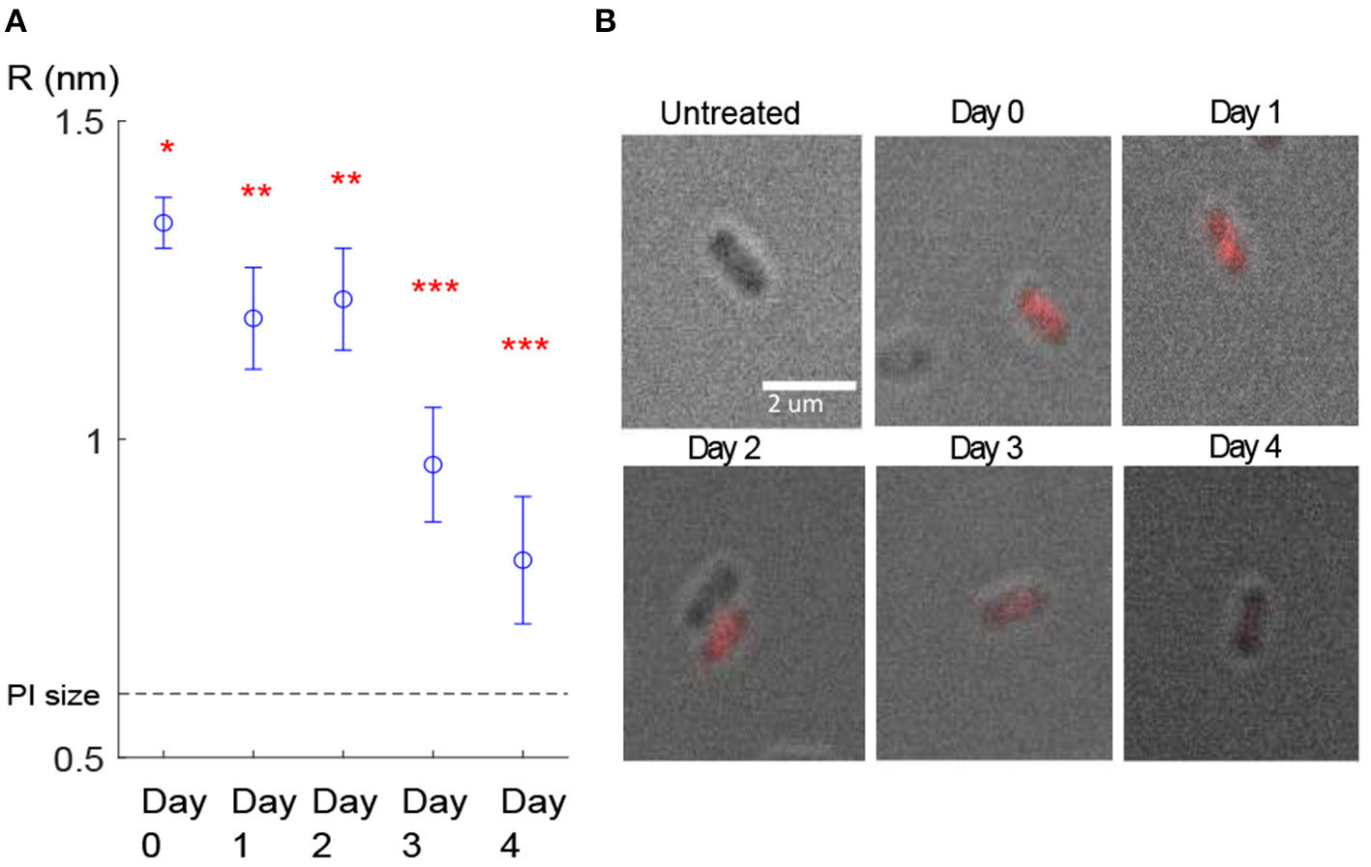
Pore size estimation. **(A)** Estimated pore radius of the cells belonging to the cluster with the lowest slope. The mean value of pore radius for day 0 was significantly (*p* < 10^−6^) different than the rest of the days. The mean value of the pore radius for days 1–2 was significantly different than days 3–4 (stars represent statistically different groups). Error bars show the confidence intervals, calculated using the Bonferroni method for multi-comparison (see 2.3). **(B)** The last frame (t = 30 min monitoring) of the fluorescence microscopy image stacks obtained in each evaluation day. Here only one cell as a representative for each day is shown.

By substituting the FI value corresponding to this cluster for each evaluation day into Equations (3–7), the mean value for pore size was estimated. First, the results of the model calibration showed that the mean radius of the pore decreased from 1.3 to 0.8 nm over the 4 days (Figure 4A).

Second, we ran one-way ANOVA and multi-comparison tests using the Bonferroni method (see section 2.3) and found that the mean value of the estimated pore radius on day 0 (mean = 1.338, SD = 0.0014) was significantly (*p* < 10^−6^) higher than on the other days (Figure 4A). The mean values of the pore radius for day 1 (mean = 1.191, SD = 0.0039) and day 2 (mean = 1.221, SD = 0.0041) were significantly different (*p* < 10^−6^) from those on days 3 (mean = 0.964, SD = 0.0043) and 4 (mean = 0.809, SD = 0.0051). Although we observed a general reduction in the PI uptake during the 4 days (Figure 4B), we did not observe a reduction from day 1 to day 2 probably owing to high variability in the results or due to other reasons such as a lag time before starting the recovery process. Furthermore, because of the small sample size on day 4 (due to a low number of PI-positive cells detected on day 4 which most likely resulted from the partial recovery of the membrane), we could not show a significant reduction from day 3 to day 4. Estimated pore size values for the cluster with the lowest slope are presented in Table 4. The decay in the pore size suggests the presence of an embedded membrane repair mechanism in bacteria, which was activated in response to HPP (see section 4). The Bartlett test reported insufficient evidence to say the variances are different with a *P*-value higher than 0.05.

**Table 4:** Estimated pore radius for the cluster with the lowest slope (Figure 4).

**Day post-pressure**	**R (nm)**	**Confidence interval^a^ (nm)**
D0	1.338	(1.296–1.380)
D1	1.191	(1.113–1.269)
D2	1.221	(1.139–1.303)
D3	0.964	(0.878–1.051)
D4	0.809	(0.702–0.915)

a *The Bonferroni method*.

**Figure 5:**
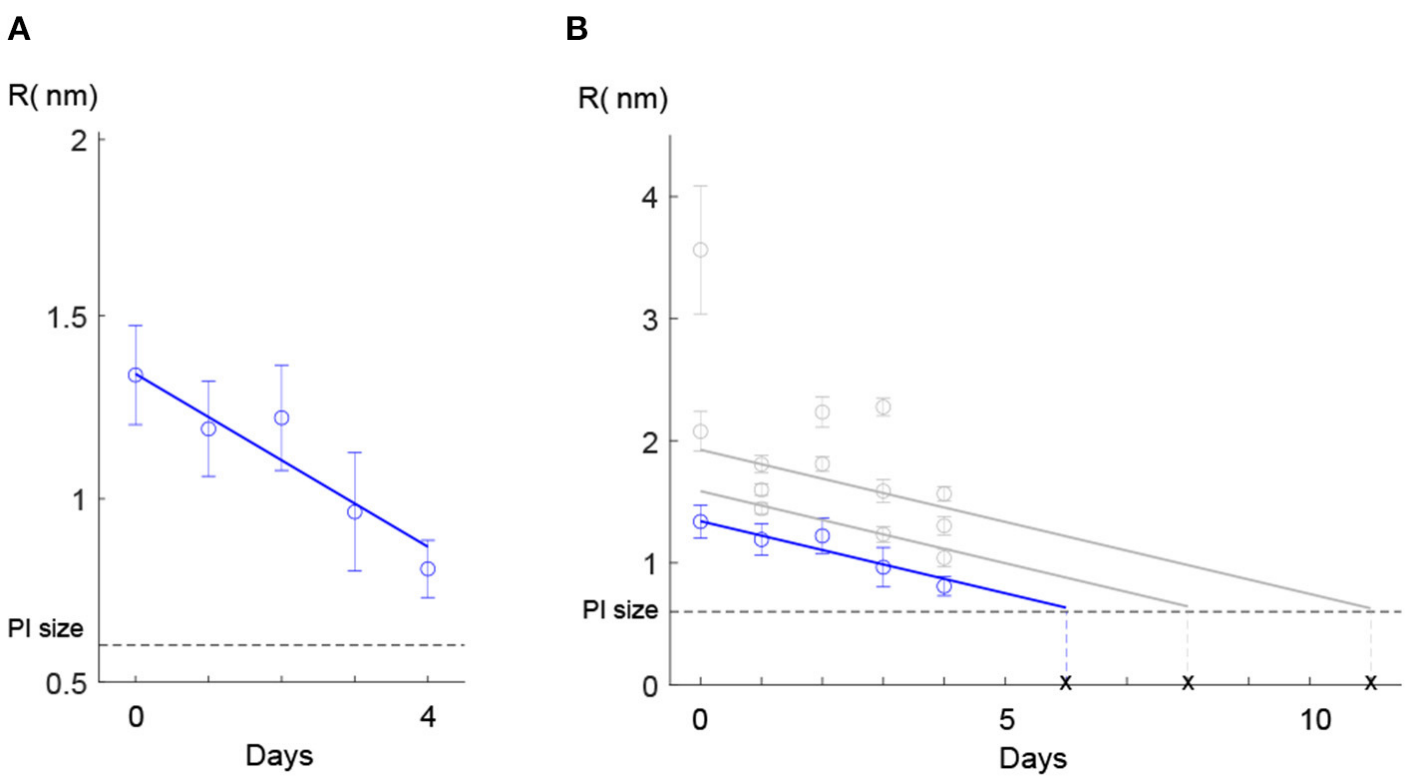
Regression model for the pore size. **(A)** A weighted regression model showed that the estimated values of the pore size for the cluster with the lowest slope in the intensity curve decayed linearly over the four days. **(B)** The linear model was fitted to the data of the remaining clusters. Error bars in both **(A,B)** show the standard deviation for the estimated pore radius. The solid blue line shows the fitted linear model to the data for the least damaged cells (the cluster with the lowest slope in the intensity curve) and the solid gray lines show the predicted linear approach for resealing pores over evaluation days for the rest of the clusters. With this linear model, we could predict the time interval needed for each group to recover the membrane.

Once the model was calibrated, we fitted a weighted least squares model to the estimated pore size values on each day for the cluster with the lowest slope (Figure 5A). Based on the assumption that cells with different pore sizes in the range <5 nm are repaired at the same rate (Zarnitsyn et al., 2008), we used the same weighted least squares model to fit the remaining cell groups (Figure 5B). Given an initial FI value on day 0, this model could predict the pore radius as a function of time. It was then possible to estimate the time that the bacterial cell needed to regain its membrane integrity. In our case, this occurred when the pore radius became smaller than the radius of the PI molecule (dashed horizontal line, Figure 5B). Although extrapolation outside the region of experimental measurements may risk entering different dynamics/regions, where the assumptions do not apply anymore, we believe that such error will not matter in our work because this is when the pores are too small to have consequences on the cell (death).

This information could be useful for food industry to design more efficient pressure treatments by adjusting HPP strategy (the pressure strength and holding time).

**Figure 6:**
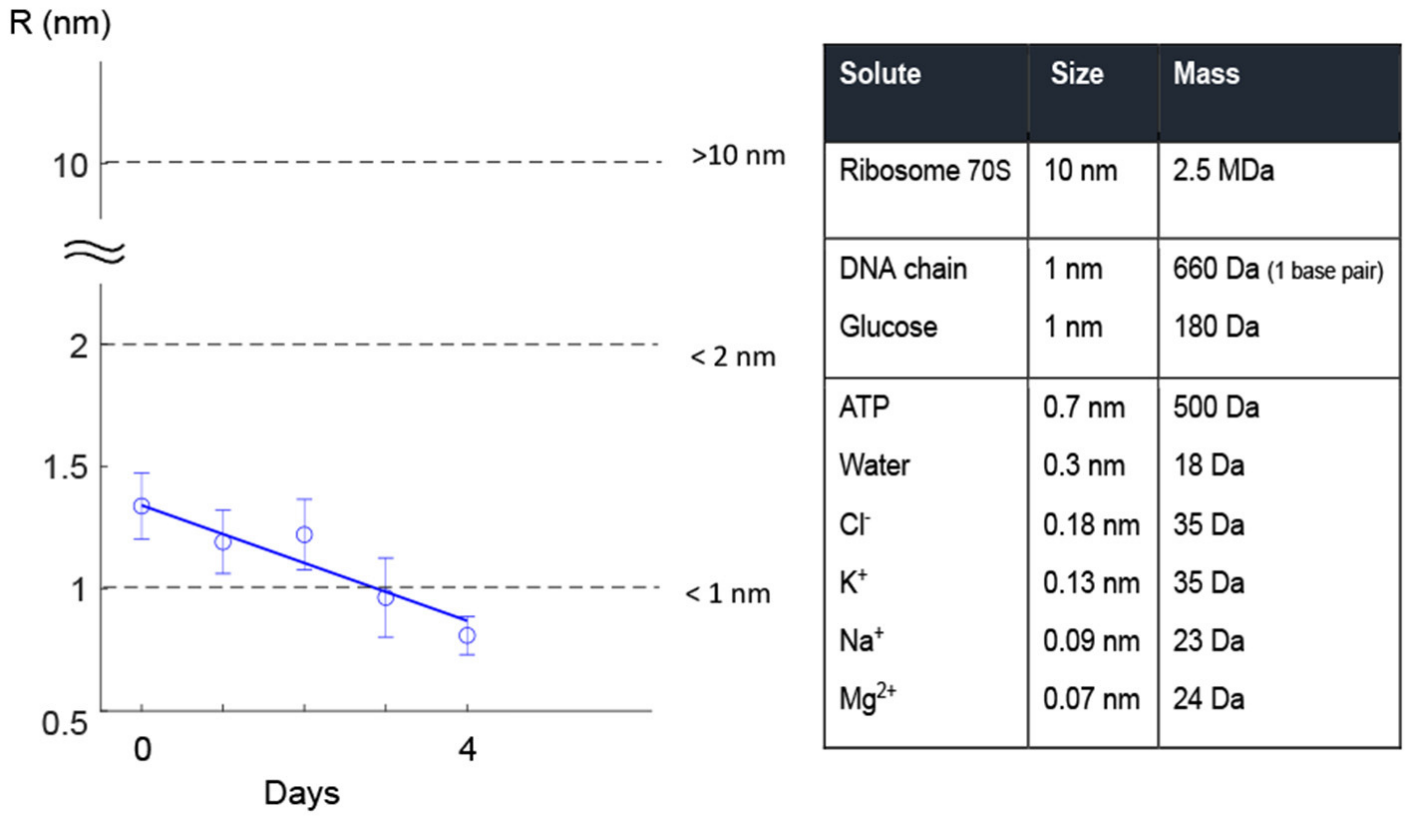
Leak of solutes through small pores. Literature research in filter membranes shows that solutes such as glucose, some ions, and ATP are small enough to pass through pores up to 1 nm size. Bigger pores may allow pass of bigger molecules such as DNA and dextrans^1^.

Figure 6 gives a better understanding of pore size in terms of allowing for the leakage of solutes with different molecule sizes through the pore. A literature search revealed that small solutes, such as ions, amino acids, ATP, etc. (size <700 Da) could pass through 1-nm pores. Larger molecules such as ribosomes and DNA were found to pass through pores larger than 2 nm (Figure 6). Although estimation of cell life expectancy requires extensive statistical experiments, it can be safely assumed that large pore sizes can cause leakage of larger essential compounds, e.g., DNA, from the cell through pores (Figure 6). In other words, the cell is more likely to die when the pore sizes are large.

To investigate how sensitive the model prediction *R* was to different parameters (e.g., *V*_*cell*_, σ, η, *h*, and *D*_*in*_), we perturbed each parameter by Δ*p* = ±10%, while the input *c*_*in*_ was 50% of its maximum value, 1 (local sensitivity analysis). We found that the model was sensitive to *V*_*cell*_, η, and *h*. The relative sensitivity coefficient ($S=|\frac{R_{\left(p\right)}-R_{\left(p+\Delta p\right)}}{\Delta p}\cdot \frac{p}{R}|$) is listed in Table 5, where *p* is the parameter and Δ*p* is its perturbation.

**Table 5:** Relative sensitivity coefficient for 10% perturbation in each parameter.

**Parameter**	**Relative sensitivity **|*S*|****
*V*_*cell*_	0.3799
η	0.3798
*h*	0.3798
σ	9.758*10^−6^
*D*_*in*_	3.188*10^−5^

**Figure 7:**
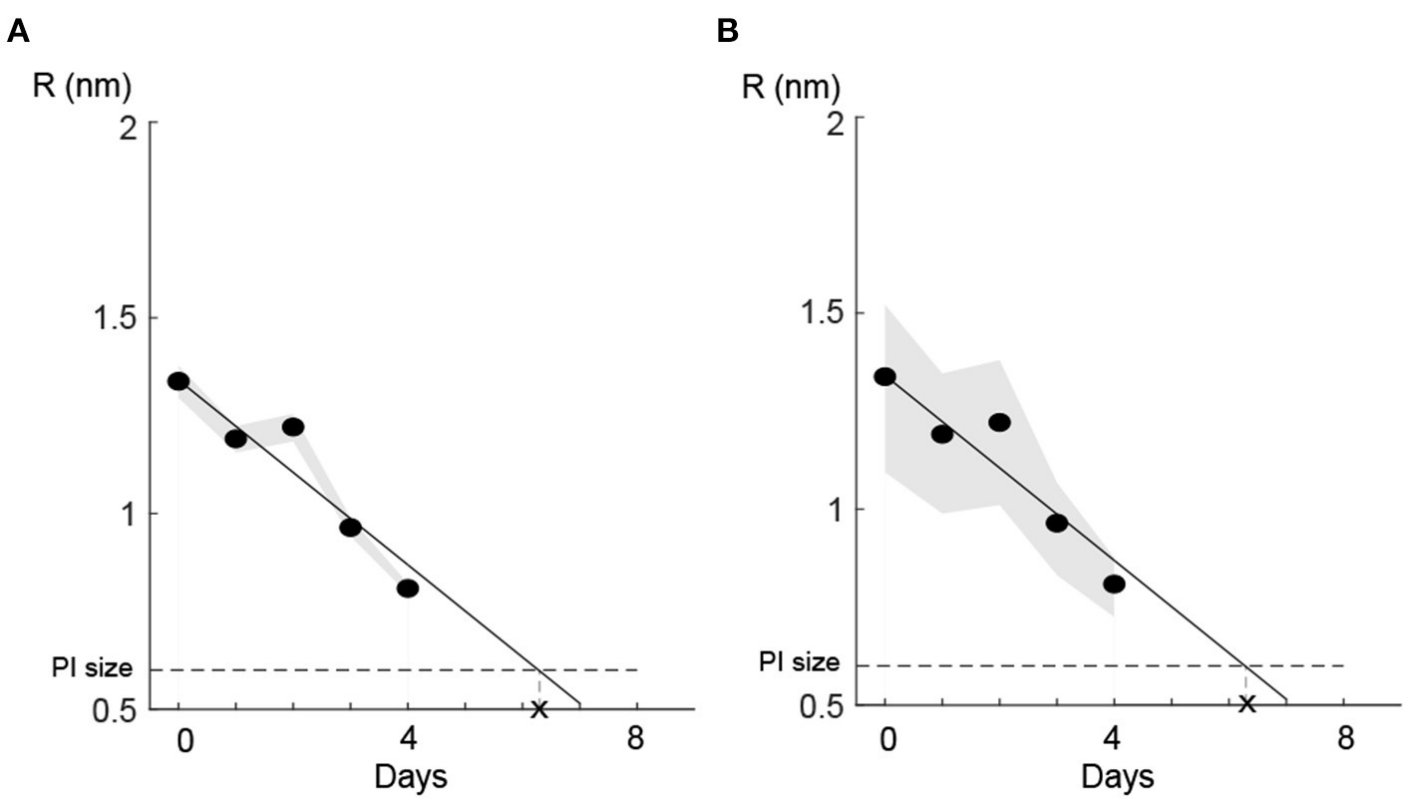
Parametric uncertainty of the linear prediction for the cell volume (Table 5) diminished with pore size. **(A)** 10% perturbation and **(B)** 50% perturbation in $p=V_{cell}=0.7*10^{-18}\left(m^{3}\right)$. Shaded bounds show the deviation of the predicted R values from its nominal values (black solid line) due to parameter (*V*_*cell*_) uncertainty. For both perturbations of 10 and 50% of the nominal parameter value, the uncertainty region diminished as pores were resealed.

To evaluate how sensitive our linear regression model was to variations in the cell volume (i.e., the most sensitive parameter in our model; Table 5), we perturbed the parameter *V*_*cell*_ (Δ*p* = ±10% and ±50%) and checked the deviation in the predicted value *R* each day. Importantly, for both Δ*p* = ±10% and Δ*p* = ±50%, the local sensitivity of the regression model to the perturbation decreased over time (Figure 7), such that the estimated time for regaining the membrane integrity did not alter significantly. We also examined the other two sensitive parameters, i.e., membrane thickness *h* and viscosity of the plasma membrane η, and found similar results. These results together with the fact that the size of *L. monocytogenes* is in the range defined by 10 and 50% perturbation from the size of *E. coli* (Jamshidi and Zeinali, 2019) indicate that the model predictions of temporal recovery were robust to the uncertainty of parameters such as *V*_*cell*_ (which was substituted from values specified for *E. coli*, Table 2).

### 3.3. Population Growth Behavior of Pressure-Treated *L. monocytogenes*

**Figure 8:**
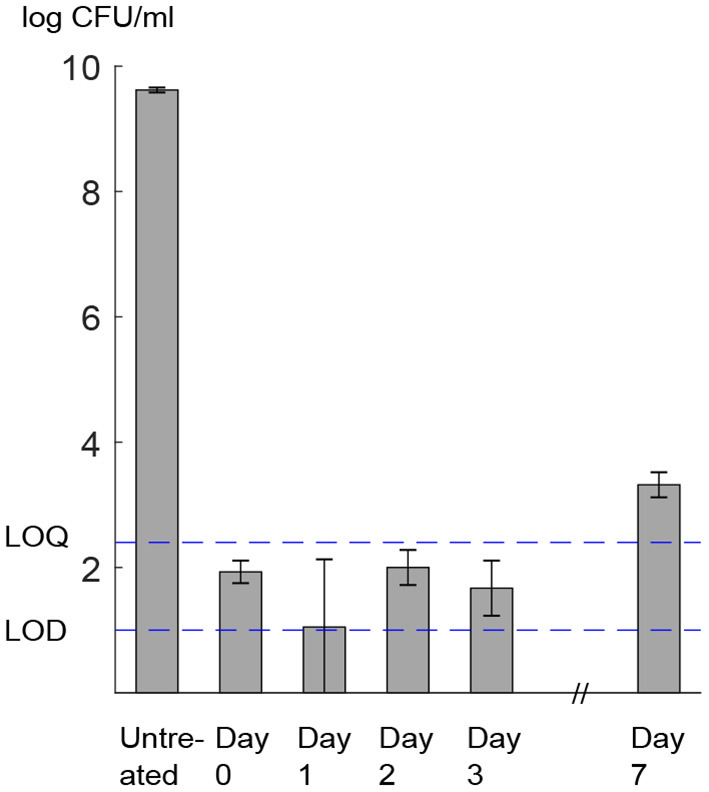
*L. monocytogenes* viable cell counts (log *CFU*/*mL*). Assessment was done by plating on non-selective medium before (untreated) and after HPP (day 0) and during storage at 8°C (day 1–7). LOD, limit of detection; LOQ, limit of quantification. Error bars show standard deviation of three biologically independent replicates. Raw data is available in Supplementary File 2.

Absence of growth at the population level during the recovery period was confirmed by measurement of viable plate counts. Exposure of *L. monocytogenes* to 400 MPa, 8 min, 8°C led to a 7.79 ± 0.82 *log* CFU/mL decrease in viable cell counts, corresponding to a cell concentration below the LOQ of the method (Figure 8). Viable cell counts in the pressure-treated sample remained constant and below the LOQ during the subsequent 4 days, whereas the growth of the total population was only observed 7 days after HPP. This trend is compatible with the existence of a lag phase of at least 4 days, followed by the onset of the exponential growth phase.

## 4. Discussion

We exposed *L. monocytogenes* cells to HPP of 400 MPa, 8 min, 8°C. Our results showed that some of the cells in the sample that were exposed to HPP became permeable to PI molecules, which otherwise could not pass through the intact membrane, suggesting the formation of pores in the cellular envelope under high pressure. Although the exact mechanism of pressure-induced membrane permeabilization is not known, it has been linked to the denaturation of membrane proteins, as well as to the phase transition of membrane phospholipids from the physiological liquid-crystalline phase to the gel phase, which results from lateral compression and increased packing of the acyl chains (Pagán and Mackey, 2000; Casadei et al., 2002; Winter and Jeworrek, 2009; Patterson, 2014). To evaluate the degree of membrane damage, we measured the rate of FI change inside each cell after adding PI molecules to the extracellular medium. First, we detected large variations in the degree of membrane damage among single cells, which were clustered into groups (Figure 3) using the k-means algorithm (see section 2.2). This was consistent with a study by Ritz et al. (2001), in which HPP followed by PI staining and flow cytometry analysis of *L. monocytogenes* revealed a broad distribution of red FIs for cells taking up PI, probably arising from different structural strengths of the cellular envelope. Entry of a bacterial population into the stationary phase, as analyzed in this study, is related to an increased resilient cell envelope and the synthesis of stress response proteins (Casadei et al., 2002; Huang et al., 2014). However, this phenotype is subject to various possible drivers of intrapopulation heterogeneity, including stochasticity in gene expression, the effects of which are amplified for low-abundance molecules such as mRNAs and regulatory proteins; cell cycle and aging stage; and epigenetic regulation (Avery, 2006; Veening et al., 2008). Moreover, phenotypic heterogeneity is an inherent feature of bacterial populations and, most importantly, is considered to be a component of their adaptation and survival strategy (Booth, 2002).

Second, the synergy of our mass transfer mechanistic model (Equations 1–7) with specially tailored experiments to test the membrane recovery hypothesis allowed us to estimate the total size of the pressure-created pores at any time point after treatment at 400 MPa, 8 min, 8°C. Our observations indicated that the rate of diffusion of PI, particularly in the cluster with the lowest slope (corresponding to the least-damaged cells), was reduced during the 4 days after exposure (Figure 4). The existence of a recovery process in the bacterial membrane investigated in this work is consistent with the results of our previous works (Duru et al., 2021; Nikparvar et al., 2021), where by using a time-series RNA sequencing data and conducting a network component analysis we proved the presence of a repair process in the membrane after HPP.

Most importantly, we found that the estimated pore size decayed (most likely) linearly as a function of time (Figure 5). If the trend is not linear, then the rate of repair (decay of size) must be increased (e.g., log decay) or decreased (e.g., exponential decay). We see no reason for an accelerated repair because cells will likely mobilize most of their resources to repair damage when it is at its maximum level. There is no reason to assume that the cells will increase their repair resources (and thus the rate) as the pore size decreases. An exponential decay (decelerating rate of repair) is otherwise more likely, but as pores are becoming smaller, the repair process will likely proceed faster. However, at that stage the cells likely divert resources to other essential damaged sites than the membrane, and therefore the rate remains nearly linear. The linear repair rate is consistent with a previous study of membrane recovery in human cells (Zarnitsyn et al., 2008), in which the authors measured membrane wound closure using several fluorescent molecules. They found an exponential decay for wound sizes on the scale of hundreds of nanometers over time, followed by an approximately linear decay for wound sizes less than 20 nm.

The model suggested in this work for quantifying the membrane damage is based on several assumptions (see 2.4). The assumption that the main pressure-induced membrane damage occurred in a single effective pore area (total area of membrane pores) may affect the prediction of the size of individual pores. To date, pores smaller than fluorescent molecules (such as PI) are difficult to measure by direct methods. Thus, the model predictions may appear biased when the number of small pores (smaller than PI size) is large. Importantly, the total diffusion of molecules through the cell membrane is only dependent on the total surface of the pores (Zarnitsyn et al., 2008), so cell fate, which is strongly affected by the total diffusion via the loss of cytosol material, will not be affected by the wrong estimation of the number of pores. Additionally, there is no evidence supporting that high pressure causes multiple small pores: once high pressure damages the membrane structure in a certain area, additional pressure buildup will most likely concentrate around the same area which is structurally weaker than the remaining parts of the membrane. Finally, finer resolution of pore sizes can be estimated by using several fluorescent molecules as in a previous work (Zarnitsyn et al., 2008) the authors compensated for this assumption (single pore area) by using a series of fluorescent molecules with different sizes. The linear relationship between the concentration of PI molecules and the FI is another fundamental assumption in our model development. This assumption may limit the accuracy of the result if saturation occurs i.e., when the increase in the PI concentration does not affect the intensity anymore. However, because we used heat-treated bacteria as positive control cells (see 2.1.3), in which the membrane damaged was highest, we guaranteed that for each day the intensity in pressure-treated bacteria was not saturated.

The intensity curves for the cluster with the lowest slope (Figure 3) displayed an increasing trend until reaching a near-saturation level. However, as the intensity approaches saturation, the sensitivity of the signal decreases. An inaccurate saturation value may cause to underestimate the pore size through Equation (5), particularly for days 0–2. For days 3 and 4, the intensity curve for the lowest-slope cluster stayed at a steady state after 30 min and therefore the estimation of the pore size for these 2 days is more accurate. However, the underestimation of the pore size on days 0–2 does not affect our conclusion of the existence of a recovery process, because even if the actual pore sizes for days 0–2 were larger than the estimated values, the damage was still recovered over days 3 and 4.

Leakage of intracellular components due to membrane damage following various treatments with antibiotics, bacteriocins, or high pressure was demonstrated for both Gram-positive and Gram-negative bacteria. In an early study, leakage of low-molecular-weight solutes upon treatment of *E. coli* with different concentrations of 2-phenoxyethanol was proposed as an indicator of the disorganization of the cytoplasmic membrane (Gilbert et al., 1977). In line with this, membrane damage induced by poly-hexamethylene biguanides resulted in leakage of potassium ions and inorganic phosphates in *E. coli* (Broxton et al., 1983). Lambert and Hammond (1973) showed leakage of potassium to be a primary indicator of membrane damage. Depending on the extent of membrane damage, larger solutes such as ATP or DNA (500–700 Da) are also released from the cell (Zhen et al., 2013; Singh et al., 2016). Although bacteria possess membrane repair mechanisms, the duration and the extent of membrane damage (i.e., pore size) may lead to bacterial cell death (Wortman and Bissonnette, 1988; Vigouroux et al., 2020). As the model developed in this work predicts the timely repair of pores, it could be integrated with future work to estimate the cumulative probability of survival and life expectancy.

We did not observe a marked increase in the number of cells able to form colonies in the culture medium during the 4 days of the experiments, i.e., cell counts remained stable and below the LOQ. However, as we could observe a decay in the membrane pore radius by time, we inferred that the growth process (mass accumulation and division) of these individual cells was arrested, yet they were not dead. Although a population growth curve does not provide information about the physiological state of cells giving rise to the exponential growth phase, single-cell studies have revealed that the presence of a non-growing fraction of cells is the main cause of the extended apparent population lag when stress conditions at the growth limit are applied (Koutsoumanis, 2008; Aguirre and Koutsoumanis, 2016), as in the present study. Moreover, several studies have shown an increased length of the lag phase, both at the population and single-cell levels, after exposure of *L. monocytogenes* to stress conditions, including high pressure (Robinson et al., 1998; Francois et al., 2006; Muñoz-Cuevas et al., 2013). The increased time required to start division can be attributed to the metabolic processes needed for repair of damaged cell components and is therefore indicative of the presence of sub-lethally injured cells (Guillier et al., 2005; Métris et al., 2008). In our experiments, cell counts exceeded the LOQ only after 7 days. Thus, it is likely that the cells that resealed their membrane pores started to proliferate and, along with a small fraction of non-injured cells (Aguirre and Koutsoumanis, 2016), contributed to the observed growth at the population level. Our observation of growth arrest at the first days after pressure treatment led us to hypothesize that for each day the cells in the group with the lowest slope came either from the cells belonging to the lowest-slope group in previous days or injured cells from other groups such as mild-slope group that transferred to the lowest-slope group as they managed to recover partially. Therefore, we knew that the less number of PI-positive cells obtained over the 4 days could not be due to proliferation of new cells but likely due to an increasing number of recovered cells.

We note that it is impossible to infer the rate of membrane damage repair from the microscopy observations alone, because of the large biological variations and the number of cell clusters (Figure 3). It was the synergy of our dynamic model, trained by our experimental data and tested on a subset of experiments, that enabled us to calculate the repair rate of any pore size on this scale (<20 nm) until the cellular envelope was repaired. This could help to predict the duration time of growth arrest after exposure to HPP until the cells restart the growth process. It should be noted that the results obtained in this work were specifically related to the pressure treatment of *L. monocytogenes* in 400 MPa, 8 min, 8°C, thereby generalizing the result to other HPP conditions and other microorganisms may not be valid. However, what we presented in this study may pave the road for future works such that the method suggested here can be applied for other HPP conditions or other microorganisms and fluorescent molecules. Repeating similar experiments with different pressure values and holding times can increase the fidelity of the model.

## 5. Conclusion

The recovery process in bacteria after exposure to high pressure has not been investigated well. Here, we focused on foodborne pathogenic bacteria *L. monocytogenes* and the effects of high pressure (400 MPa, 8 min, 8°C) on its membrane. We added PI molecules to the pressure-treated bacteria at time point 0 (immediately after treatment) and on days 1, 2, 3, and 4 after HPP, and measured the FI emitted by DNA-bound PI molecules using a fluorescence microscopy technique. We developed a dynamic model to quantify the degree of damage in pressure-treated bacteria. The synergy between our diffusion model and microscopy experiments revealed that some *L. monocytogenes* cells exposed to HPP repaired their damaged membrane approximately linearly on a time scale of days. This is the first time that membrane pores created by HPP have been quantitatively described and shown to diminish.

## Data Availability Statement

The original contributions presented in the study are publicly available. This data can be found here: https://datadryad.org/stash/share/bP2iD9QM03-5tCl8njy_0BNrcQAHgX5dZvCwznZwmvw.

## Author Contributions

BN: conceptualization, methodology, software, formal analysis, investigation, and writing—original draft, visualization. AS: methodology, investigation, and writing—original draft. MC: resources, writing—review and editing, and supervision. MH-H: resources and supervision. PC: visualization and writing—review and editing. CR: writing—review and editing and supervision. NB: conceptualization, validation, formal analysis, investigation, resources, writing—review and editing, supervision, and project administration. All authors contributed to the article and approved the submitted version.

## Conflict of Interest

The authors declare that the research was conducted in the absence of any commercial or financial relationships that could be construed as a potential conflict of interest.

## Footnotes

^1^London Health Science Centre (2020). https://www.lhsc.on.ca/critical-care-trauma-centre/principles-of-crrt/ (accessed July 24, 2020).

## Abbreviations

*E. coli*: *Escherichia coli*
FI: Fluorescence intensity
HPP: High pressure processing
*L. monocytogenes*: *Listeria monocytogenes*
LOD: Limit of detection
LOQ: Limit of quantification
PI: Propidium iodide.
